# The Social Determinants of Infant Mortality and Birth Outcomes in Western Developed Nations: A Cross-Country Systematic Review

**DOI:** 10.3390/ijerph10062296

**Published:** 2013-06-05

**Authors:** Daniel Kim, Adrianna Saada

**Affiliations:** 1 Behavioural and Policy Sciences Department, RAND Corporation, 20 Park Plaza, Suite 920, Boston, MA 02116, USA; 2 Department of Social and Behavioural Sciences, Ecole des Hautes Etudes en Santé Publique, Rennes 35043, France; 3 Center for Health Decision Science, Harvard School of Public Health, Boston, MA 02115, USA; E-Mail: adrianna.saada@gmail.com

**Keywords:** social determinants of health, infant mortality, birth outcomes, preterm birth, United States, Western Europe

## Abstract

Infant mortality (IM) and birth outcomes, key population health indicators, have lifelong implications for individuals, and are unequally distributed globally. Even among western industrialized nations, striking cross-country and within-country patterns are evident. We sought to better understand these variations across and within the United States of America (USA) and Western Europe (WE), by conceptualizing a social determinants of IM/birth outcomes framework, and systematically reviewing the empirical literature on hypothesized social determinants (e.g., social policies, neighbourhood deprivation, individual socioeconomic status (SES)) and intermediary determinants (e.g., health behaviours). To date, the evidence suggests that income inequality and social policies (e.g., maternal leave policies) may help to explain cross-country variations in IM/birth outcomes. Within countries, the evidence also supports neighbourhood SES (USA, WE) and income inequality (USA) as social determinants. By contrast, within-country social cohesion/social capital has been underexplored. At the individual level, mixed associations have been found between individual SES, race/ethnicity, and selected intermediary factors (e.g., psychosocial factors) with IM/birth outcomes. Meanwhile, this review identifies several methodological gaps, including the underuse of prospective designs and the presence of residual confounding in a number of studies. Ultimately, addressing such gaps including through novel approaches to strengthen causal inference and implementing both health and non-health policies may reduce inequities in IM/birth outcomes across the western developed world.

## 1. Introduction

### 1.1. Between-Country Variations in IM/Birth Outcomes

**Figure 1:**
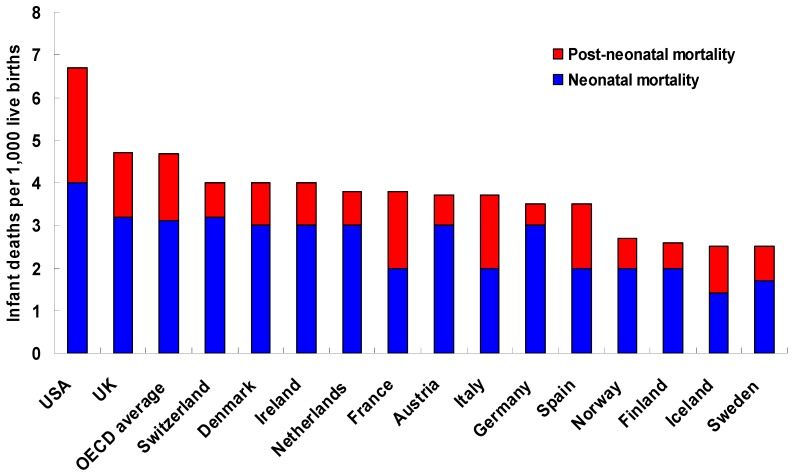
Infant mortality rates (number of infant deaths per 1,000 live births) in 2008 in selected OECD member countries. *Source*: OECD Health Data 2010 (April 2011 version) and WHO Global Health Observatory 2011.

Infant mortality (IM), an important health outcome during the first year of life, is unequally distributed across countries at a global level [1]. Among Organization for Economic Co-operation and Development (OECD) countries, in 2008, infant deaths per 1,000 live births ranged from a low of 1.8 in Luxembourg to a high of 15.2 in Mexico [2]. Although advances in medicine and public health in the western industrialized world over the course of the 20th century produced major reductions in aggregate infant mortality rates (IMR), the United States (USA) ranks poorly compared to most other high income economies [2,3], with an IMR of 6.7 deaths per 1,000 live births in 2008 [2]. IMRs in the Western European (WE) nations of the United Kingdom (UK), France, Germany, and Nordic countries ranged from 2.5 to 4.7 deaths per 1,000 live births (Figure 1) [2].

Cross-national variations in birth outcomes are equally apparent in the fetal mortality rate (FMR), which includes spontaneous intrauterine deaths occurring at ≥20 weeks of gestation. Annual fetal deaths account for almost as much reproductive loss as IM in the USA, with a FMR of 6.2 fetal deaths per 1,000 live births and fetal deaths in 2005 [4]. Slightly lower FMRs were observed for WE countries in 2000 [5]. Neonatal mortality, or death occurring at ≤28 days of age, shows similar patterns. The 2006 USA neonatal mortality rate (NMR) was 4.5 deaths per 1,000 live births [6]. In 2000, the NMR was ≤3 deaths per 1,000 live births in each of France, Germany, and Nordic countries and 4 deaths per 1,000 live births in the UK [5].

**Figure 2:**
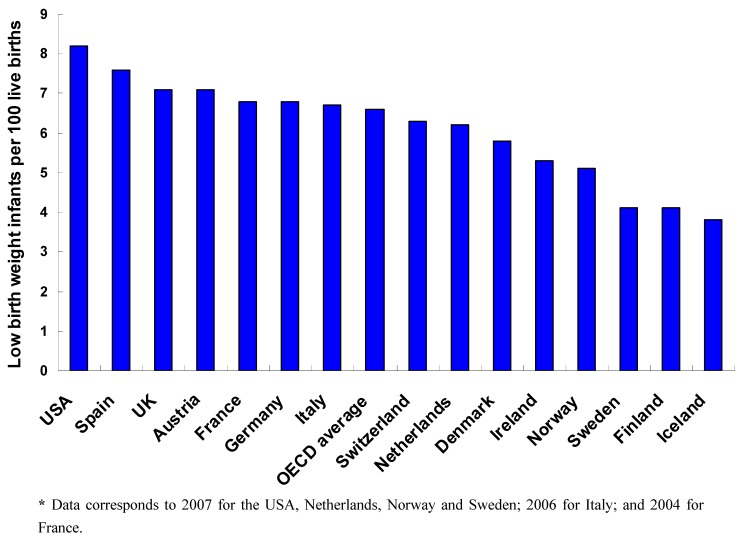
Infant low birth weight rates (number of low birth weight infants per 100 live births) in 2008 in selected OECD member countries.* Source: OECD Health Data 2011 (April 2011 version), World Bank 2011 (World Development Indicators), and WHO Regional Office for Europe (Health for All Database).

Preterm birth (PTB) (<37 weeks gestation) and very preterm birth (VPTB) (<32 weeks gestation) are leading causes of mortality and morbidity in infants worldwide [7,8,9]. In 2005, 7.5% of all births in developed countries were preterm. PTB rates were lowest in Europe (6.2%) and highest in North America (10.6%) [7]. Meanwhile, 12.2% of all USA births were preterm in 2009—a significant decrease since 2006 [10]. PTB is closely linked to low birth weight (LBW) and very low birth weight (VLBW)—infants weighing less than 2,500 grams and 1,500 grams, respectively. Globally, LBW infants have a 20 times higher risk of death than heavier infants [9]. LBW infants account for 8.2% of all live births in the USA and 4.8% to 7.1% of all live births in WE countries (Figure 2) [11].

IM and birth outcomes are key population health indicators, for three primary reasons. First, IM is a widely accepted indicator of social development and economic change [12], and IM/birth outcomes enable comparisons of population health attainment across diverse settings. Second, these outcomes are thought to be sensitive to even short-term broad changes in social and economic conditions and health care. Third, birth outcomes signify important lifelong implications for the health, social, and economic outcomes in individuals [13], and IM carries public health significance based on the potential years of life lost (PYLL).

### 1.2. Within-Country Variations in IM/Birth Outcomes

Wide variations in birth outcomes are also observed within western developed nations. Racial/ethnic disparities in IM are persistent within the USA, with Blacks carrying an excessive burden of infant death that has a significant impact on overall national trends [3,14]. Socioeconomic disparities in birth outcomes are additionally pervasive [15]. Other individual level factors such as maternal health behaviours [16] and psychosocial stress [17] may further contribute to differential pregnancy outcomes.

Disproportionate levels of IM/adverse birth outcomes are present within WE nations. The UK exhibits large variations in IM among different ethnic groups [18,19]. PTB rates vary widely geographically, and are particularly high in the UK’s Northern and Trent regions [20]. Differences in PTB rates also exist among ethnic groups in France [21,22] and Germany [23], yet geographical variations in IMR appear to be relatively small [24]. Similar patterns of social inequalities in fetal/perinatal mortality are observed in Nordic countries, although less consistently [25].

### 1.3. A Social Determinants of IM/Birth Outcomes Conceptual Framework

To better understand these striking cross-country and within-country patterns, a conceptual framework for the societal to individual level determinants of IM/birth outcomes is needed. In particular, identifying contextual social determinants at the upstream macro level may help to explain the wide variations in IM/birth outcomes *across* countries. Likewise, a social determinant of IM/birth outcomes framework which conceptualises the dynamic interplay between contextual and individual level social determinants with IM/birth outcomes may aid in deciphering patterns of disparities in IM/birth outcomes *within* countries.

**Figure 3:**
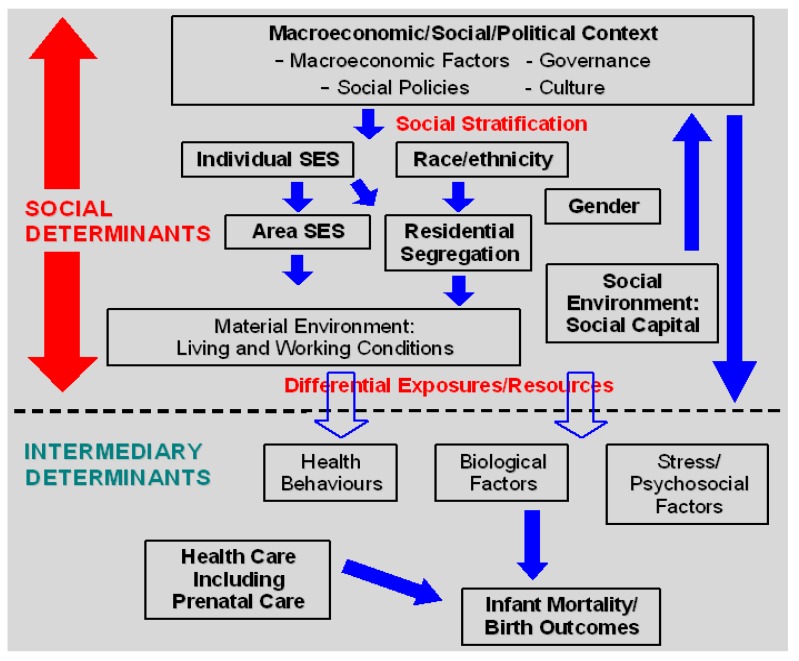
A Social Determinants of IM/Birth Outcomes Conceptual Framework. Adapted from Solar & Irwin [27].

Drawing upon work of the World Health Organisation’s Commission on Social Determinants of Health [26,27], we offer an adapted conceptual framework with a focus on the hypothesized *social determinants of IM/birth outcomes* (Figure 3). As seen in this figure, the social determinants consist of the *material living and working conditions* and *social environmental conditions in which people are born*, *live*, *work*, *and age*, and the *structural drivers* of these conditions, comprised of individual and area level socioeconomic status (SES), race/ethnicity, residential segregation, gender, social capital/cohesion, and the macroeconomic and macrosocial context, e.g., macroeconomic and social policies including labour market regulations [28], political factors including governance and political rights [29,30], and culture. Macroeconomic determinants include the gross domestic product (GDP) *per capita* and income inequality. The broader macroeconomic and social context generates social stratification *i.e.*, the sorting of people into dominant and subordinate SES, racial/ethnic, and gender groups (Figure 3). Through stratification and differential exposures of individuals to levels of material factors/social resources, social determinants such as individual/area level SES, race/ethnicity, and social capital shape individual level intermediary determinants, including behavioural factors (e.g., maternal smoking), biological factors, and psychosocial factors (e.g., social support), which in turn produce differential risks of, and inequities in, IM/birth outcomes (Figure 3). 

Access to health care and quality of health care are also determinants of these outcomes, but may play lesser roles compared to other societal factors (Figure 3). For example, following Medicaid expansions for pregnant women in the USA, between 1986 and 1993, rates of low birth weight significantly declined among White women of low SES compared to during the preceding period [31]. Other studies have not found that expanding health insurance coverage to uninsured low income pregnant women or earlier initiation of prenatal care is associated with improvements in birth outcomes [32,33]. Furthermore, access to prenatal care may be influenced by social determinants including individual SES and neighborhood material conditions such as access to transportation [34]. 

While not depicted in the figure, time is an additional inherent element of the framework. That is, each of these social determinants, intermediary determinants, and IM/birth outcomes exists within populations and individuals at specific points in time, and their causal relationships with one another are sensitive to the time that separates them e.g., current neighbourhood (area level) SES influences future health behaviours at some, but not other, points in time in women over the lifecourse; these behaviours in turn shape future IM/birth outcomes.

## 2. Methods

### 2.1. Systematic Literature Review

We systematically reviewed the empirical literature on each of these contextual and individual level social determinants (with the exception of health care) of IM/birth outcomes, within and across western developed nations *i.e.*, USA and WE states. To our knowledge, this represents the first comprehensive review of the social determinants of IM/birth outcomes. We searched PubMed, EmBase, and PsychInfo databases from 1966 to 31 December 2011, using combinations of keywords/subject headings to identify original articles and systematic review articles of birth outcomes and cross-country macroeconomic and macrosocial social determinants, within-country contextual social determinants, and within-country individual level social determinants: “birth outcomes”, “infant mortality”, “fetal mortality”, “neonatal mortality”, “preterm birth”, “low birth weight”, “social determinants”, “gross domestic product”, “income inequality”, “social policies”, “maternity leave”, “neighbourhood deprivation”, “neighbourhood socioeconomic status”, “maternal socioeconomic status”, “race/ethnicity”, “residential segregation”, “social cohesion/capital”, “maternal health behaviour”, “maternal smoking”, “maternal stress/distress”. For the purpose of this systematic review, we focused on non-medical social and economic determinants of birth outcomes, and did not review health care/systems as a social determinant. Recent national and international commissioned reports on the social determinants of health, including by the WHO Commission on the Social Determinants of Health [26] and the Robert Wood Johnson Foundation Commission to Build a Healthier America [35], have likewise placed primary emphasis on non-medical societal determinants across a range of health outcomes. Criteria for inclusion were English-language studies conducted on the USA, UK, Austria, Belgium, Denmark, Finland, France, Germany, Greece, Iceland, Italy, Luxembourg, Netherlands, Norway, Portugal, Spain, Sweden, and Switzerland. Based on abstracts returned from our search, we reviewed relevant papers, and searched their references for additional papers. We limited our review to studies on the USA and WE states, and excluded studies on developing nations, in order to ensure higher data quality and reduce confounding due to uncontrolled/unmeasured factors. For studies included for each key social determinant, we then noted the observed direction (compared to the hypothesized direction) and statistical significance of findings (using a 5% significance level). 

This study addresses a significant gap in the literature to date: that so far there has been insufficient attention paid to the social determinants of IM/birth outcomes as compared to adult health, including in wealthy/industrialized nations. Furthermore, while several systematic reviews of selected social determinants in relation to birth outcomes have been published, no work to date has yet considered them simultaneously. By doing so, we aimed to identify global patterns and gaps in the literature on the social determinants of IM/birth outcomes, and thereby to advance research in these areas. Through promoting understanding of the ways in which societal determinants may facilitate or hinder health and well-being at the very start of life, we may ultimately inform more effective interventions and policies to reduce health inequalities across the lifecourse in the western developed world.

## 3. Results

For each social determinant, Table 1 classifies studies into the following categories according to their results: significant in the expected direction (positive or inverse, as indicated in Table 1), significant in the opposite direction than expected, mixed (significant and nonsignificant) findings for different indicators of the same construct or for similar outcomes, and null findings. Studies are listed within each category in reverse chronological order.

### 3.1. Cross-Country Evidence on Macroeconomic and Macrosocial Determinants

#### 3.1.1. Macroeconomic Determinants

##### Gross Domestic Product (GDP)

Country level economic development, commonly measured using GDP *per capita*, appears to influence IM, at least below a certain threshold or during earlier time periods. Of 140 references returned in our search and the articles cited in relevant publications, three original articles met the inclusion criteria (Table 1). Rodgers [36] demonstrated that GDP *per capita* (reflecting average standards of living for households) is linked ecologically and cross sectionally to IMRs across developing and developed countries, with a curvilinear relationship suggesting diminishing health returns to higher GDP among rich nations. In a time series analysis of high and middle income countries (including the USA, UK, and France) that examined changes in exposures and outcomes (“first difference models”) and thereby reduced confounding, Ensor and colleagues [37] found a modest yet significant inverse association between GDP growth *per capita* and IMRs between 1936 and 1965; this association was absent in more recent time periods (after 1965). Similar patterns among 18 OECD countries were seen by Wennemo [38].

##### Income Inequality

Of 2,116 references yielded in our search and references cited in relevant abstracts, 14 individual studies (identified through one systematic review and eight other original articles) satisfied the inclusion criteria (Table 1). Income inequality, the distribution of income within populations shaped by macroeconomic policies, may help to account for cross-national variations in IM/birth outcomes. Possible mechanisms include underinvestments in public goods such as health care and education, and the adverse effects of relative deprivation on maternal psychosocial factors. Of 14 epidemiological studies, nine ecological, cross sectional studies with varying degrees of adjustments for country level factors showed significant positive associations between higher income inequality and IMRs in western industrialized nations and across a range of developed and developing countries [38,39,40,41,42,43,44,45,46]. The other five ecological studies found small, nonsignificant relationships between economic inequality and IMRs [47,48,49,50,51]. For LBW, two of three ecological studies showed positive linkages [40,41,42].

**Table 1:** Distribution of studies of the relations between social determinants and IM/birth outcomes, by direction and significance of findings.

Social determinants		Studies with significant findings in expected direction (POS = positive association, INV = inverse association with IM/LBW/PTB)	Studies with significant findings in opposite direction than expected	Studies with mixed findings (significant and nonsignificant)	Studies with null findings	Total number of studies
**Cross-country macroeconomic** ** and macrosocial determinants**	Gross domestic product *	1 IMR (INV):	-	2 IMR:	-	**3 (IMR)**
		Rodgers, 1979 [36]		Ensor *et al.*, 2010 [37]; Wennemo, 1993 [38]		
	Income inequality *	9 IMR (POS):	-	-	5 IMR:	**14 (IMR)**
		Macinko *et al*., 2004 [39]; Muntaner *et al*., 2002 [40]; Lynch *et al*., 2001 [41]; Ruhm, 2000 [42]; Hales *et al*., 1999 [43]; McIsaac & Wilkinson, 1997 [44]; Wennemo, 1993 [38]; Waldmann, 1992 [45]; Pampel & Pillai, 1986 [46]			Leigh *et al*., 2007 [47]; Kennelly *et al*., 2003 [48]; Wildman *et al*., 2003 [49]; Mellor & Milyo, 2001[50]; Judge *et al*., 1998 [51]	**3 (LBW)**
		2 LBW (POS) Muntaner *et al*., 2002 [40]; Lynch *et al*., 2001 [41]			1 LBW: Ruhm, 2000 [42]	
	Social policies *	4 IMR/NMR (INV):	-	1 IMR/LBW:	-	**5 (IMR/LBW)**
		Heymann *et al*., 2011 [52]; Bradley *et al*., 2011 [53]; Filmer & Pritchett, 1999 [54]; Wennemo, 1993 [38]		Pampel & Pillai, 1986 [46]		
**Within-country contextual social determinants**	Neighbourhood SES/material conditions	**USA (INV):**	-	**USA:**	**USA:**	**USA: 31** **WE: 18**
		*21 multilevel*		*5 multilevel*	*2 multilevel*	
		Janevic *et al*., 2010 [55]; Holzman *et al*., 2009 [56]; O’Campo *et al*., 2008 [57]; Williams *et al*., 2007 [58]; Currie & Moretti, 2007 [59]; Masi *et al*., 2007 [60]; Farley *et al*., 2006 [61]; Grady, 2006 [62]; Messer *et al*., 2006 [63]; Subramanian *et al*., 2006 [64]; Krieger *et al*., 2005 [65]; Reagan *et al*., 2005 [66]; Buka *et al*., 2003 [67]; Krieger *et al*., 2003 [68]; Rich-Edwards *et al*., 2003 [69]; Kaufman *et al*., 2003 [70]; Pickett *et al*., 2002 [71]; Wegner *et al*., 2001 [72]; Rauh *et al*., 2001 [73]; Fang *et al*., 1999 [74]		Messer *et al*., 2008 [89]; Collins *et al*., 2006 [90]; Ahern *et al*., 2003 [91]; English *et al*., 2003 [92]; Pearl *et al*., 2001 [93]	Hillemeier *et al*., 2007 [97]; Morenoff *et al*., 2003 [98]	
		*1 ecological*		*1 ecological*	*2 ecological*	
		Silva *et al*., 2001 [75]		Howell *et al*., 2005 [94]	Young *et al*., 2010 [99]; Jaffee & Perloff, 2003 [100]	
		**WE (INV):**		**WE:**	**WE:**	
		*12 multilevel*		*2 multilevel*	*3 multilevel*	
		Sundquist *et al*., 2011 [76]; Agyemang *et al*., 2009 [77]; Sellström*et al*., 2007 [78]; Dibben *et al*., 2006 [79]; Janghorbani *et al*., 2006 [80]; Lasbeur *et al*., 2006 [81]; Smith *et al*., 2006 [82]; Bundred *et al*., 2003 [83]; Aveyard *et al*., 2002 [84]; Bonellie, 2001 [85]; Spencer *et al*., 1999 [86]; Spencer *et al*., 1999 [87]		Taylor-Robinson *et al*., 2011 [95]; Zeitlin *et al*., 2011 [96]	Calling *et al.*, 2011 [101]; Clausen *et al.*, 2006 [102]; Delpisheh *et al*., 2006 [103]	
		*1 ecological*				
		Smith *et al*., 2007 [88]				
**Within-country contextual social determinants**	Residential segregation	**USA (POS):**	**USA:**	-	**USA:**	**USA: 19** **WE: 2**
		*4 multilevel*	*2 multilevel*		*1 multilevel*	
		Debbink & Bader, 2011 [104]; Kramer *et al*., 2010 [105]; Walton, 2009 [106]; Bell *et al*., 2006 [107]	Vinikoor *et al*., 2008 [120]; Bell *et al*., 2006 [107]		Hearst *et al*., 2008 [122]	
		*12 ecological*				
		McFarland & Smith, 2011 [108]; Lobmayer & Wilkinson, 2002 [109]; Guest *et al*., 1998 [110]; Polednak, 1996 [111]; Bird & Bauman, 1995 [112]; LaVeist, 1993 [113]; Polednak, 1993 [114]; Polednak, 1991 [115]; Laveist, 1990 [116]; LaVeist, 1989 [117]; Yankauer & Allaway, 1958 [118]; Yankauer, 1950 [119]	**WE:**			
			*2 multilevel*			
			Zeitlin *et al*., 2010 [96]; Pickett *et al*., 2009 [121]			
	Income inequality	**USA (POS):**	-	-	**USA:**	**USA: 11** **WE: 1**
		*1 multilevel*			*1 multilevel*	
		Nkansah-Amankra *et al*., 2010 [123]			Finch, 2003 [131]	
		*7 ecological*			*2 ecological*	
		Olson *et al*., 2010 (IMR, LBW) [124]; Sohler & Arno, 2003 (IMR) [125]; Lobmayer & Wilkinson, 2002 (IMR) [109]; Ross *et al*., 2000 (IMR) [126]; Shi *et al*., 1999 (IMR, LBW) [127]; Kennedy *et al*., 1996 (IMR) [128]; Kaplan *et al*., 1996 (LBW) [129]			Deaton & Lubotsky, 2003 (IMR) [132]; Mellor & Milyo, 2001 (IMR, LBW) [50]	
		**WE:**				
		*1 ecological*				
		Materia *et al*., 2005 [130]				
	Social cohesion/social capital	**USA (INV):**	-	**USA:**	-	**USA: 2**
		*1 ecological*		*1 multilevel*		
		Kawachi *et al*., 1997 [133]		Buka *et al*., 2003 [67]		
**Within-country individual level social determinants**	Race/ethnicity **	**USA (POS for non-White groups *vs.* Whites): 19**	-	**USA: 5**	**USA: 5**	**USA: 29** **WE: 9**
		Nabukera *et al*., 2009 [134]; Shen *et al*., 2008 [135]; Ehrenthal *et al*., 2007 [136]; Kistka *et al*., 2007 [137]; Buescher & Mittal, 2006 [138]; Dominguez *et al*., 2005 [139]; Dole *et al*., 2004 [140]; Rich-Edwards *et al*., 2003 [69]; Rosenberg *et al*., 2002 [141]; Berg *et al*., 2001 [142]; Adams *et al*., 2000 [143]; Foster *et al*., 2000 [144]; Alexander *et al*., 1999 [145]; David & Collins, 1997 [146]; Singh & Yu, 1996 [147]; Schoendorf *et al*., 1992 [148]; Abrams & Newman, 1991 [149]; Kleinman & Kessel, 1987 [150]; Shiono & Klebanoff, 1986 [151]		Collins *et al*., 2004 [156]; Mustillo *et al*., 2004 [157]; Rauh, 2001 [73]; Collins *et al*., 2000 [158]; Goldenberg *et al*., 1998 [159]	Dailey, 2009 [160]; Reagan & Salsberry, 2005 [66]; Korte, 1999 [161]; Shiono *et al*., 1997 [162]; Murrell, 1996 [163]	
		**WE (POS for non-White groups *vs.* Whites): 8**		**WE: 1**		
		Reeske *et al*., 2011 [152]; Gray *et al*., 2009 [18]; Kelly *et al*., 2008 [153]; Zeitlin *et al*., 2004 [21]; Patel *et al*., 2003 [19]; Essén *et al*., 2000 [154]; Vangen *et al*., 2002 [155]; Zeitlin *et al*., 1998 [22]		Aveyard, 2002 [84]		
**Within-country individual level social determinants**	Individual SES **	**USA (INV): 28**	-	**USA: 7**	**USA: 9**	**USA: 44** **WE: 23**
		Acevedo-Garcia * et al*., 2007 [164]; Astone* et al*., 2007 [165]; El Reda* et al*., 2007 [166]; Williams *et al*., 2007 [58]; Masi *et al*., 2007 [60]; Colen* et al*., 2006 [167]; Farley *et al*., 2006 [61]; Goldman *et al*., 2006 [168]; Grady, 2006 [62]; Madan* et al*., 2006 [169]; Messer* et al*., 2006 [170]; Subramanian *et al*., 2006 [64]; Acevedo-Garcia* et al*., 2005 [171]; Ponce* et al*., 2005 [172]; Nicolaidis* et al*., 2004 [173]; Savitz* et al*., 2004 [174]; Steward & Moser, 2004 [175]; Gould* et al*., 2003 [176]; Jaffee, 2003 [100]; Rich-Edwards *et al*., 2003 [69]; Pickett *et al*., 2002 [71]; Abrevaya, 2001 [177]; Pearl *et al*., 2001 [93]; Rauh *et al*., 2001 [73]; Rolett & Kiely, 2000 [178]; Shmueli & Cullen, 2000 [179]; Fang *et al*., 1999 [74]; Gorman, 1999 [180]		Blumenshine *et al*., 2011 [193]; Reagan *et al*., 2007 [194]; Reagan, 2005 [66]; Finch, 2003 [195]; Braveman *et al*., 2001 [196]; Conley & Bennett, 2001 [197]; Parker *et al*., 1994 [198]	Currie & Moretti, 2007 [59]; Hillemeier *et al*., 2007 [97]; Dominguez, 2005 [139]; Kaufman *et al*., 2003 [70]; Morenoff, 2003 [98]; Misra *et al*., 2001 [203]; Conley & Bennett, 2000 [197]; Foster *et al*., 2000 [144]; Longo *et al*., 1999 [204]	
		**WE (INV): 14**		**WE: 4**	**WE: 5**	
		Dibben *et al*., 2006 [79]; du Prel* et al*., 2006 [181]; Gisselmann, 2006 [182]; Reime* et al*., 2006 [183]; Fairley, 2005 [184]; Thompson* et al*., 2006 [185]; Spencer* et al*., 2004 [186]; Gissler* et al*., 2003 [187]; Ronda & Regidor, 2003 [188]; Moser *et al.*, 2003 [189]; Raum *et al.*, 2001 [190]; Spencer *et al.*, 1999 [86]; Ancel *et al.*, 1999 [191]; Basso *et al.*, 1999 [192]		Nobile *et al*., 2007 [199]; Voight *et al*., 2004 [200]; Grimmer *et al*., 2002 [201]; Lekea-Karanika *et al*., 1999 [202]	Sellström, 2007 [78]; Villalbi *et al*., 2007 [205]; Dejin-Karlsson & Ostergren, 2004 [206]; Skórzyńska & Rudnicka-Drozak, 1999 [207]; Vagero *et al*., 1999 [208]	
**Within-country individual level social determinants**	Health behaviours **	**USA (POS):**	-	-	**USA:**	**USA: 11** **WE: 9**
		*6 smoking (prospective)*			*5 smoking (prospective)*	
		Lobel *et al*., 2008 [209]; Orr *et al*., 1996 [210]; Doucette & Bracken, 1993 [211]; Shiono *et al*., 1986 [212]; van den Berg & Oechsli, 1984 [213]; Frazier *et al*., 1961 [214]			Siega-Riz *et al.*, 1996 [221]; Wen *et al.*, 1990 [222]; Naeye, 1982 [223]; Rush & Kass, 1972 [224]; Yerushalmy, 1964 [225]	
		**WE (POS):**			**WE:**	
		*6 smoking (prospective)*			*3 smoking (prospective)*	
		Wisborg *et al*., 1996 [215]; Henriksen *et al*., 1995 [216]; Ahlborg & Bodin, 1991 [217]; Stein *et al*., 1987 [218]; Obel, 1979 [219]; Russell *et al*., 1968 [220]			Nordentoft *et al*.,1996 [226]; Peacock *et al*., 1995 [227]; Donovan, 1977 [228]	
**Within-country individual level social determinants**	Maternal psychosocial factors **	**USA (POS):**	-	-	**USA:**	**USA:** **10 stress,** **15 depression** **WE:** **7 stress,** **7 depression**
		*6 stress (prospective)*			*4 stress (prospective)*	
		Glynn *et al*., 2008 [229]; Lobel *et al*., 2008 [209]; Stinson & Lee, 2003 [230]; Orr *et al*., 2002 [231]; Wadhwa *et al*., 1993 [232]; Reeb *et al*., 1987 [233]			Kramer *et al*., 2009 [246]; Neggers, 2006 [235]; Strange, 2004 [247]; James, 2000 [248]	
		*6 depression (prospective)*			*9 depression (prospective)*	
		Wisner *et al*., 2009 [234]; Neggers *et al*., 2006 [235]; Jesse *et al*., 2003 [236]; Orr et al.,, 2002 [231]; Zimmer-Gembeck & Helfand, 1996 [237]; Steer *et al*., 1992 [238]			Diego *et al*., 2009 [249]; Gavin *et al*., 2009 [250]; Li *et al*., 2009 [251]; Suri *et al*., 2007 [252]; Haas *et al*., 2005 [253]; Dole *et al*., 2003 [254]; Hoffman & Hatch, 2000 [255]; Copper *et al*., 1996 [256]; Perkin *et al*., 1993 [257]	
		**WE (POS):**			**WE:**	
		*4 stress (prospective)*			*3 stress (prospective)*	
		Class *et al*., 2011 [239]; Martini *et al*., 2010 [240]; Khashan *et al*., 2009 [241]; Hedegaard *et al*., 1996 [242]			Krabbendam *et al*., 2005 [258]; Nordentoft *et al*., 1996 [226]; Henriksen *et al*., 1994 [259]	
		*3 depression (prospective)*			*4 depression (prospective)*	
		Dayan *et al*., 2006 [243]; Dayan *et al*., 1999 [244]; Hedegaard *et al*., 1993 [245]			Elsenbruch *et al*., 2007 [260]; Berle *et al*., 2005 [261]; Andersson *et al*., 2004 [262]; Nordentoft *et al*., 1996 [226]	

* All ecological studies. ** All individual level studies. Statistical significance was defined by a *p value* <0.05 (where reported).

#### 3.1.2. Macrosocial Determinants

##### Social Policies

Of 1,665 references and articles cited, five original articles were included in our review (Table 1). Social policies, particularly those structuring maternal leave programs, may be key determinants of IM/birth outcomes through improving the quality of prenatal care and adult care to neonates/infants [52]. In the USA, working women are entitled to ≥12 weeks of *unpaid* maternity leave through the 1993 Family and Medical Leave Act [263]. By contrast, WE countries have implemented *paid* maternity leave policies since 1945. In WE countries, paid maternity leave ranges from a maximum of 14 weeks in Germany and Switzerland to approximately 77 weeks in Sweden [2]. Contemporary policies aim to prevent PTB by granting women time off and offering generous financial compensation (90–100% of salary). The initiation of maternity leave varies by WE country: 6–8 weeks pre-delivery in France and Germany, 10 weeks pre-delivery in Sweden, and 12 weeks pre-delivery in the UK. Financial compensation of salary during maternity leave is likewise generous: women are compensated at 100% in France and Germany, and at 90% in Sweden and Denmark. Countries such as France have achieved improvements in maternity leave policies and documented downward trends in PTB rates over the past 30 years, although it is difficult to show a causal link between the two because population wide policies eliminate the possibility of controls for evaluation [264]. Nevertheless, a recent cross-national, cross sectional study among 141 OECD and non OECD countries found that an increase of 10 weeks of paid maternal leave predicted 10% significantly lower NMRs and IMRs, controlling for important covariates [52].

Government spending on non-health factors, and to a lesser extent on health factors, may also help to account for cross-country variations in IMRs. In a pooled cross sectional analysis adjusting for multiple country level factors, total government spending (medical care, public health, social welfare) was significantly inversely associated with post-neonatal mortality rates (for deaths 29 days to one year of age), but not NMRs [46]. In a recent cross sectional study, Bradley and colleagues [53] found that the ratio of social to health expenditures was significantly protective against IMRs, controlling for GDP *per capita*. Two other investigations, including one that used instrumental variable analysis, also supported a greater role of non-health *versus* health spending [38,54].

### 3.2. Within-Country Evidence on Contextual Social Determinants

#### 3.2.1. Neighbourhood SES/Material Conditions

Based on 513 references and articles cited in relevant papers, 49 studies (31 USA, 18 WE studies, identified through one systematic review and 11 other original articles) met the inclusion criteria (Table 1).

*USA*. Neighbourhood level socioeconomic deprivation may partially account for variations in USA pregnancy outcomes through enabling women’s access to material resources and services. We identified 31 studies of neighbourhood SES and birth outcomes in the USA (Table 1). The majority of studies (21 of 31) found significant associations between a neighbourhood SES indicator or index (*i.e.*, neighbourhood and area level income, poverty, education, employment, occupation, housing, and residential stability) and an adverse birth outcome (*i.e.*, PTB and/or LBW) (Table 1). Significant positive associations in five studies were specific to a racial/ethnic subgroup [55,63,70,71,74]. Meanwhile, only five [55,59,66,67,71] of the 31 studies analyzed data from a prospective/retrospective cohort, all of which had significant findings, while the majority of studies used cross sectional designs; four studies were ecological [75,94,99,100]. All studies adjusted for age, and all five cohort studies controlled for parental SES and race/ethnicity either through statistical adjustment or stratification. No studies in the literature have yet examined the associations between availability of specific material goods/services within neighbourhoods and IM/birth outcomes.

*WE*. Neighbourhood socioeconomic deprivation also appears to determine birth outcomes in Western Europe. We identified 18 studies of neighbourhood SES and IM/birth outcomes (Table 1). Most (13 of 18) studies found significant associations between neighbourhood SES and IM/birth outcomes. Six [76,83,87,95,101,102] of the 18 studies analyzed prospective/retrospective cohort data, of which three studies had significant findings [76,85,86]. Eleven studies used cross sectional study designs [77,78,79,80,81,82,83,84,86,96,103] and one study was ecological [88]. All studies adjusted for age. However, only one study [84] in countries with ethnically heterogeneous populations controlled for both parental SES and race/ethnicity.

#### 3.2.2. Residential Segregation

Of 35 references and articles cited in those publications, 21 individual studies (19 USA, two WE) were included (Table 1).

*USA*. Residential segregation, defined as the extent to which social groups characterized by income or race/ethnicity are spatially separated from one another, may also contribute to IM/birth outcome disparities through the effects of harmful material and psychosocial environments within segregated communities. In an ecological analysis, residential segregation *by income* was positively related to IMRs, independent of mean household income and metropolitan area income inequality [109]. To date, all 12 ecological studies on residential segregation *by race/ethnicity* have found associations with higher IM/PTB risks [108,109,110,111,112,113,114,115,116,117,118,119]. Of seven multilevel, multivariate studies, four studies observed significant associations for racial segregation among Blacks [104,105,106,107]. Another multilevel analysis used propensity score methods to reduce confounding, and found no effect of racial segregation on IM [122]. Two other studies identified protective associations for racial segregation [107,120]. Living in racially homogeneous neighbourhoods may protect against IM/birth outcomes through the “ethnic density effect”, *i.e.*, the benefits from residing in a neighbourhood containing same-ethnic individuals as oneself, possibly through political empowerment and social cohesion (as indicated by the arrow going from residential segregation to the social environment/social capital in Figure 3) [107,120].

*WE*. Racial/ethnic segregation levels are generally lower in European cities than USA cities [73]. This may account for the lack of investigation of impacts of residential segregation on IM/birth outcomes in WE nations. The closest related (though not synonymous) concept studied in WE is same-ethnic density. In two multilevel, cross-sectional studies, same-ethnic density had marginally protective associations against PTB for foreign-born (but not native) women in France [96] and for Pakistani women (but not women of other ethnicities) in the UK [121], possibly through the ethnic density effect.

#### 3.2.3. Income Inequality

Of 2,116 references and articles cited in relevant papers, 12 studies (11 USA, one WE original articles) satisfied our inclusion criteria (Table 1).

*USA.* Six [109,124,125,126,127,128] of 8 ecological, cross sectional studies [50,109,124,125,126,127,128,132] on state/metropolitan area income inequality and IMRs that primarily adjusted for area level income showed significant positive relationships; three [124,127,129] of four studies [50,124,127,129] on income inequality and LBW found positive associations. Only two studies have been multilevel: higher neighbourhood level income was linked to a higher individual risk of LBW for Blacks, independent of maternal income [123]. Controlling for state and individual level covariates, Finch [131] found no independent effect of state level income inequality on the individual probability of IM.

*WE.* Few studies in WE have examined the associations between income inequality and IM/birth outcomes. One ecological study in Italy observed a positive relation between the provincial Gini coefficient and IMRs [130].

#### 3.2.4. Social Cohesion/Social Capital

Of 13 references and articles cited in those publications, two original articles (both from the USA) were included in the review (Table 1).

*USA*. The degrees of social support, trust, networks, and connectedness characterizing a neighbourhood or community are referred to as stocks of social capital/cohesion [265], and could influence health through social support, diffusion of knowledge on healthy behaviours, and/or collective action leading to policies that provide health promoting public goods [266]. In an ecological, cross sectional study, lower state level social capital (trust, associational memberships) was strongly linked to higher IMRs in Blacks [133]. In a multilevel, cross sectional analysis, low perceived neighbourhood cohesion predicted lower infant birth weight among Blacks but not Whites [67].

*WE*. There is a dearth of research on social cohesion and infant health in WE nations, such that no studies were identified on this topic.

### 3.3. Within-Country Evidence on Individual Level Social Determinants

Of 91 references and articles cited, 38 articles (29 USA and nine WE studies, identified through two systematic reviews and 17 other original articles) met the inclusion criteria (Table 1).

*USA*. Recent USA data shows a more than two-fold difference between non-Hispanic Blacks and non-Hispanic Whites for the IMR (12.7 *vs.* 5.5 deaths per 1,000 live births), NMR (9.0 *vs.* 3.6), FMR (11.1 *vs.* 4.8), and PMR (12.3 *vs.* 5.6) [6,226,259]. These racial/ethnic disparities have been framed through a variety of socioeconomic, behavioural, biological, and genetic explanatory lenses [267,268,269]. Race may also determine IM/birth outcomes through racial residential segregation. Nineteen of 29 USA-based studies have found positive linkages between race/ethnicity and VPTB or VLBW after adjusting for individual level factors (Table 1). Other research identifies certain antecedents of PTB (e.g., maternal age, multiple gestation births) and LBW (e.g., unhealthy lifestyle behaviours, inadequate prenatal care) as contributing factors to racial/ethnic disparities [270]. However, even in low risk populations, there is strong evidence for racial/ethnic variation in birth outcomes [144,145]. Racial discrimination may further play a role. In a recent systematic review of 10 studies of racial discrimination and risks of PTB, LBW, and VLBW, Giurgescu *et al*. [271] found three studies with positive and significant associations, three studies with mixed significant and nonsignificant findings, and four studies with null associations.

*WE*. Increases in migration to WE nations in recent decades have affected birth outcomes of ethnic populations. The risks of fetal, neonatal, and infant mortality are generally higher among refugees and non-refugee migrants than non migrants, although these patterns vary by country of origin and receiving country [272]. Findings from three UK studies suggest that women from certain ethnic groups (e.g., Black African, Black Caribbean, Asian, Indian, Pakistani, and Bangladeshi) have higher adjusted risks of adverse birth outcomes than White women [26,27,153]. French women of Afro-Caribbean origin experience the highest PTB rates [29] and those of Sub Saharan African origin have the greatest odds of perinatal mortality [30]. Migrants from the Middle East and North Africa (including Turkey) in Germany have significantly higher risks of fetal death than non-migrants [152]. Foreign born women in Nordic countries have relatively higher adjusted risks of perinatal mortality [154], higher FMRs, and poorer birth outcomes [155].

#### 3.3.2. Individual SES

Of 1,808 references and articles cited in relevant papers, 67 studies (44 USA and 23 WE studies, identified through one systematic review and three other original articles) were included in our review (Table 1).

*USA.* Parental SES may account for variations in IM/birth outcomes by shaping access to instrumental resources for adopting healthy practices and avoiding harmful risks; it also sorts individuals into different socioeconomic environments e.g., neighbourhoods of residence. We identified 44 American studies of parental SES and birth outcomes (Table 1). Twenty eight of these studies found significant inverse associations between ≥1 socioeconomic indicator and an adverse birth outcome, although in 11 studies the associations were specific to a population subgroup. Of the 44 studies, only 10 studies [59,66,70,139,144,165,167,174,194,197] (three with significant findings; [165,167,174]) used data from a prospective cohort, while the remainder of studies employed case-control or cross sectional study designs. All but one cohort study [194] adjusted for age, while all but two studies [139,165] controlled for race/ethnicity.

*WE*. Parental SES is also a key predictor of birth outcomes in WE nations. We identified 23 studies of parental SES and birth outcomes (Table 1). Fourteen of the studies observed significant associations. Twenty studies relied on case-control or cross sectional study designs, whereas only three studies [86,190,206] were based on a prospective cohort, with significant findings in two studies [86,190]. Two of the three cohort studies adjusted for maternal age [190,206]; one [190] of two cohort studies [86,190] in countries with ethnic heterogeneity controlled for race/ethnicity. For IM, a systematic review of studies published between 1980 and 2000 suggests that SES inequalities exist across Nordic countries [273]; other Nordic studies support SES linkages to IM [101,274,275].

#### 3.3.3. Health Behaviours

Of 1,902 references and articles cited in relevant papers, 20 prospective studies on maternal smoking and birth outcomes (11 USA and nine WE studies, identified through one systematic review) met the inclusion criteria (Table 1).

*USA*. Preconception health behaviours (e.g., healthy eating, regular exercise) may protect against adverse birth outcomes, while other behaviours (e.g., smoking, alcohol misuse, and inadequate intake of folic acid) may contribute to them [91]. Maternal smoking, a key prevalent modifiable risk factor during pregnancy, has been previously investigated in association with PTB in 64 studies [16]. Of 11 USA based prospective studies controlling for covariates including age, race/ethnicity, and income, six studies linked maternal smoking to significantly higher PTB odds, while findings in the other five studies were null. Evidence suggests that a low glycemic Mediterranean-type diet during pregnancy may decrease PTB risk [276]. Periconceptional multivitamin use has also been significantly inversely linked to the risk of PTB [277]. However, few high-quality studies to date have explored the roles of nutrient deficiencies in PTB [278].

*WE*. The implications of poor maternal health behaviours likewise apply within WE countries. In the UK, maternal obesity has been associated with significantly higher risks of IM [279] and adverse birth outcomes [280]. Other UK studies highlight the association between alcohol consumption and early fetal death [281]. For smoking, of nine WE-based prospective studies, six studies found that maternal smoking predicts significantly higher PTB odds [16].

#### 3.3.4. Maternal Psychosocial Factors

Of 930 references and the articles cited in those publications, 39 articles (25 USA and 14 WE studies, identified through two systematic reviews) were included (Table 1).

*USA*. Maternal psychosocial factors may be important determinants of birth outcomes, plausibly acting through behavioural or direct physiologic pathways [17,282]. Differential levels of stress during pregnancy may contribute to disparities in perinatal health [283]. In six of 10 prospective USA studies, the multivariate adjusted relations between stress during pregnancy and PTB/LBW were significantly positive [17,209,229,230,231,232,233]. In a meta-analysis of 15 American prospective studies of depression during pregnancy and birth outcomes (14 studies of PTB and six studies of LBW, with six of 15 studies showing significant associations for PTB/LBW overall), Grote *et al*. [282] found significant yet modest summary adjusted relative risks (RRs) of 1.10 between antenatal depression and risks of each of PTB and LBW.

*WE*. Maternal distress and anxiety can independently contribute to adverse fetal and neonatal outcomes [284]. Of seven WE studies on the prospective relationships between antenatal psychosocial stress and PTB/LBW, four studies found significant positive associations [17,239,240,241,242]. In a meta-analysis of seven prospective studies of depression during pregnancy and birth outcomes (five PTB and three LBW studies, with three of seven studies showing significant associations for PTB/LBW overall), there were significant and nonsignificant summary RRs of 1.37 and 1.16 between antenatal depression and PTB and LBW, respectively [282].

## 4. Discussion

This paper focused on key indicators of population health at the very onset of life—IM and birth outcomes—reflecting short term and wide ranging changes in societal conditions, indicators that can signify profound social and economic sequelae over the life course for individuals and populations. To explain patterns of IM/birth outcome variations across and within western developed countries, we conceptualized a framework and conducted systematic reviews of the empirical literature on the social determinants of IM/birth outcomes, spanning determinants from the societal down to the individual level. Unlike previous systematic reviews, we considered these social determinants and intermediary factors simultaneously to identify more general patterns and gaps that characterize this literature.

Consistent with the well known curvilinear relationship between GDP and life expectancy—the Preston curve [285]—with diminishing health returns to higher GDP among rich nations, the limited evidence to date suggests GDP *per capita* may play a modest role in explaining current IM/birth outcome variations across the western developed world. Meanwhile, the epidemiological literature provides support for other macroeconomic/societal conditions as more important explanatory factors. For instance, nearly two-thirds of income inequality studies have found linkages with IM/birth outcomes, although these studies have been largely ecological and cross sectional in design. Likewise, in ecological cross sectional studies**, **paid maternal leave policies**, **levels of social spending, and ratios of social to health spending have all been found to predict IMRs in the hypothesized directions.

Within both the USA and WE countries, the evidence in roughly two-thirds of studies suggests that neighbourhood SES is a determinant of adverse birth outcomes. However, most of these studies have been cross sectional rather than prospective. Furthermore, no studies have yet explored whether specific neighbourhood material goods and services may be related to IM/birth outcomes; such analyses could help to unpack the specific mechanisms for the effects of neighbourhood SES. Most studies on residential segregation by race/ethnicity in the USA show positive findings, although have been largely ecological in design; multilevel studies which account for individual level factors exhibit more mixed findings. In WE countries, the presence of lower levels of residential segregation may account for the lack of investigation of segregation in those settings; the limited evidence to date supports a health-protective ethnic density effect of segregation. Meanwhile, social cohesion/capital has been relatively underexplored as a predictor of birth outcomes. The few published studies in the USA support a relationship, while no studies have yet been conducted on social cohesion/capital and IM/birth outcomes within WE countries. Like for neighbourhood SES, studies on individual SES have found primarily inverse associations with IM/birth outcomes, but the majority of studies have likewise been cross sectional. For other individual level social determinants, associations have been relatively mixed for race/ethnicity and selected intermediary behavioural and psychosocial factors (e.g., smoking, maternal stress and depression).

Adopting a social determinants of IM/birth outcomes conceptual framework and jointly examining the empirical evidence on these social determinants further suggests several conceptual and methodological gaps in the literature. First, our framework highlights multiple levels of social determinants, the presence of multiple factors at each level, and the importance of stratification by SES, race/ethnicity, and gender [26,27] to the production of inequities in IM/birth outcomes. Studies and reviews to date have presented, either explicitly or implicitly, generally more simplified conceptual frameworks. While a number of studies of contextual social determinants (e.g., neighbourhood SES) have incorporated a multilevel structure, as we note above, many other studies have been ecological; even in multilevel studies, there have been varying levels of adjustment for key compositional factors such as parental SES and race/ethnicity, and other social determinants at the same or higher spatial levels (e.g., social cohesion, social policies). As with other observational studies in which exposures do not vary randomly [286], concerns are raised about the presence of true associations *versus* spurious associations due to residual confounding. In future investigations, statistical models should attempt to specify other social determinants at multiple levels. Furthermore, this literature would benefit from the growing arsenal of novel analytical approaches to improve causal inference, such as instrumental variable analysis [266], propensity score methods [287], and marginal structural models [288].

Second, because social stratification leads to differential exposures to social determinants, and thereby to material conditions and psychosocial resources, some demographic and socioeconomic population groups may exhibit stronger associations for social determinants with health outcomes than other groups [289]. Yet apart from race/ethnicity (in studies of area level SES effects), compositional factors such as maternal age, SES, and rural/urban status have largely been ignored as possible effect modifiers in studies to date. By identifying such effect modifiers, future interventions and policies could be tailored towards vulnerable population groups [290], and could potentially lead to more effective reductions in IM/birth outcome disparities.

Third, among the cross-country and within-country contextual social determinants that we reviewed, nearly two-thirds (64.2%) of investigations have focused on factors at the neighbourhood level (SES, residential segregation). Critically, studies of macroeconomic and macrosocial factors at higher geographical levels than neighbourhoods comprise only about one-third (35.8%) of studies on contextual social determinants. Better understanding the social determinants of IM/birth outcome disparities and better addressing these inequities will require in depth research and attention to these most fundamental “causes of causes” of health and disease [26,291].

Notably, our study was limited in several respects. As indicated earlier, it excluded the literature on a wider set of countries, including developing nations which are characterized by a higher burden of IM/adverse birth outcomes. This exclusion was to reduce potential residual confounding, although at the price of reduced generalisability. In addition, we did not perform a meta-analysis or other quantitative analysis, in light of the heterogeneity of effect size measures, presence of cross sectional designs, and lack of adjustment for important confounders in many studies, as well as insufficient numbers of studies for some social determinants (e.g., GDP, social capital) that reduced our confidence in the validity of summary estimates [292]. We instead relied on systematic reviews to attempt to identify more general qualitative patterns for each social determinant, and patterns across social determinants. Finally, as stated at the outset, we did not examine health care/systems as a social determinant, and focused our systematic review on non-medical social and economic determinants of IM/birth outcomes.

## 5. Conclusions

In keeping with the recent Adelaide Statement on Health in All Policies [293], at a societal level, both health and non-health policies that address the social determinants of health are needed to tackle IM/birth outcome disparities [294]. Implementing such policies while addressing key research gaps for the social determinants of IM/birth outcomes may optimally reduce inequities in these vital health outcomes across and within the western industrialized world.
